# Relational grounding facilitates development of scientifically useful multiscale models

**DOI:** 10.1186/1742-4682-8-35

**Published:** 2011-09-27

**Authors:** C Anthony Hunt, Glen EP Ropella, Tai ning Lam, Andrew D Gewitz

**Affiliations:** 1 Department of Bioengineering and Therapeutic Sciences, University of California, San Francisco, CA 94143, USA; 2 Tempus Dictum, Inc., Portland, OR 97222, USA; 3 School of Pharmacy, Chinese University of Hong Kong, Shatin, NT, Hong Kong; 4 Institute for Computational and Mathematical Engineering and School of Medicine, Stanford University, Stanford, CA, 94305, USA

## Abstract

We review grounding issues that influence the scientific usefulness of any biomedical multiscale model (MSM). Groundings are the collection of units, dimensions, and/or objects to which a variable or model constituent refers. To date, models that primarily use continuous mathematics rely heavily on absolute grounding, whereas those that primarily use discrete software paradigms (e.g., object-oriented, agent-based, actor) typically employ relational grounding. We review grounding issues and identify strategies to address them. We maintain that grounding issues should be addressed at the start of any MSM project and should be reevaluated throughout the model development process. We make the following points. Grounding decisions influence model flexibility, adaptability, and thus reusability. Grounding choices should be influenced by measures, uncertainty, system information, and the nature of available validation data. Absolute grounding complicates the process of combining models to form larger models unless all are grounded absolutely. Relational grounding facilitates referent knowledge embodiment within computational mechanisms but requires separate model-to-referent mappings. Absolute grounding can simplify integration by forcing common units and, hence, a common integration target, but context change may require model reengineering. Relational grounding enables synthesis of large, composite (multi-module) models that can be robust to context changes. Because biological components have varying degrees of autonomy, corresponding components in MSMs need to do the same. Relational grounding facilitates achieving such autonomy. Biomimetic analogues designed to facilitate translational research and development must have long lifecycles. Exploring mechanisms of normal-to-disease transition requires model components that are grounded relationally. Multi-paradigm modeling requires both hyperspatial and relational grounding.

## Review

### Needed: models that bridge multiple scales of organization

A research goal (Goal 1) for computational biology, translational research, quantitative pharmacology, and other biomedical domains involves discovering and validating causal linkages between components within a biological system in both normal and pathologic settings. The translational goal (Goal 2) is to use that knowledge to improve existing and discover new therapeutic interventions. Vital to each is the formulation and implementation of computational models that, like wet-lab models, are (Goal 3) suitable objects of experimentation and represent domains in which confidence in experimental predictions is sufficient for decision making under specifiable conditions. These models, much like the systems they aim to study, must bridge multiple scales of organization, and therefore require capabilities that represent (and account for) the many uncertainties that arise in the multiscale model setting. Just as mechanistic hypotheses and insight evolve with the persistent accumulation of new wet-lab knowledge, mechanistic representations within the software constructs comprising computational models must be capable of evolving and accommodating concurrently in order to be scientifically useful. Such changes cannot be smoothly and easily achieved without prior consideration of model grounding issues at all model development stages. The purpose of this review is to provide a critical assessment of emerging technology and present arguments and examples in support of the preceding statement.

**Figure 1:**
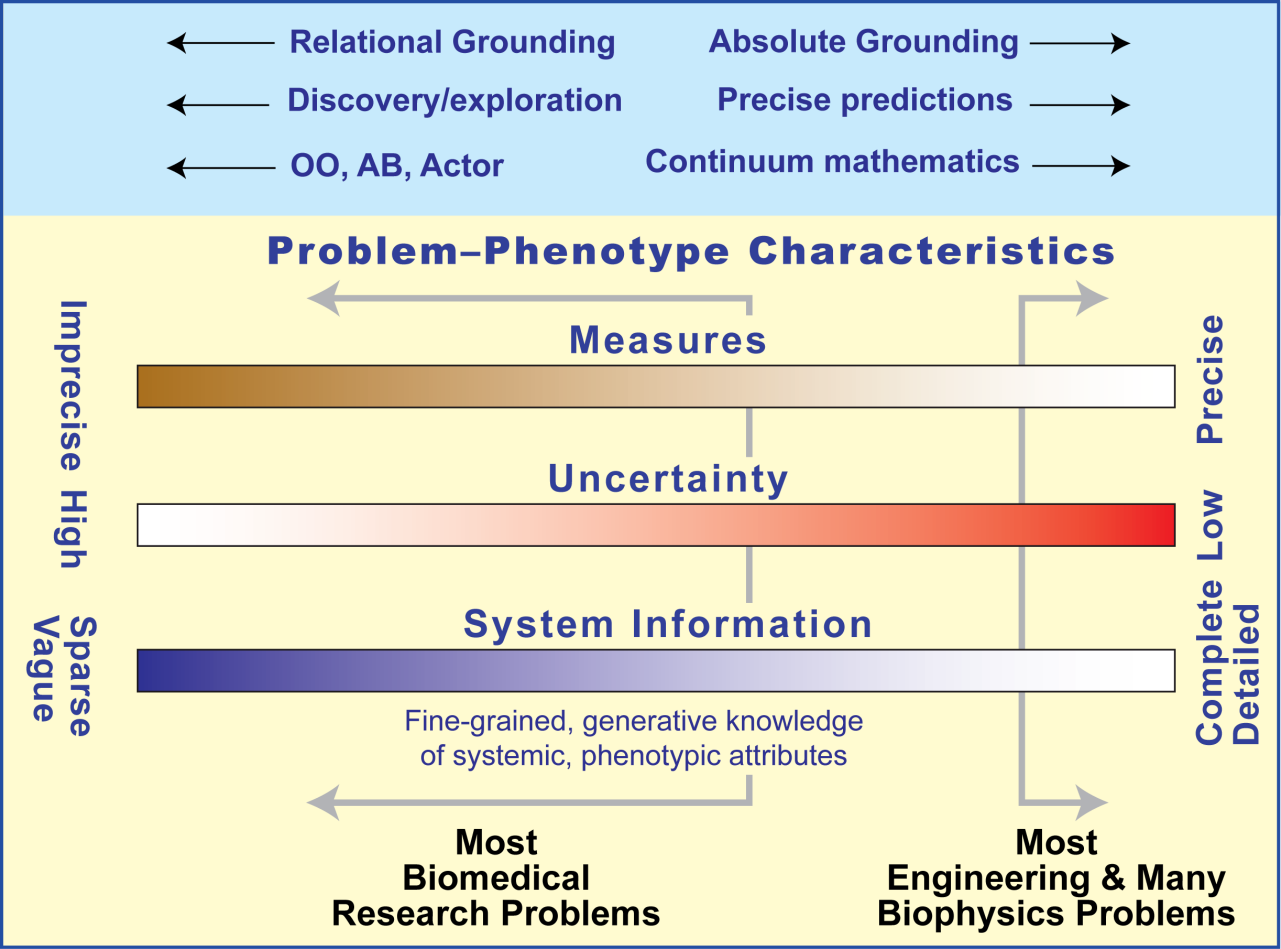
**Characteristics of scientific problem and system phenotype**.

A glossary is provided. When glossary terms are first used in the text, they are footnoted and defined under Endnotes. The units, dimensions, and/or objects to which a variable or model constituent refers establish groundings. Each term, variable, or object in a model has a meaning established by either an external context (foundational) or by other terms in the model (internal consistency). Absolute grounding*^a ^*is most prevalent in the literature; its variables, parameters, and input-output (I/O) are in real-world units like seconds and micrograms. Each term is foundational and maps to a tacit thing with an established, real-world meaning. By contrast, relational grounding*^b ^*represents variables, parameters, and I/O in units defined by other system components. Terms are defined in terms of each other in an internally consistent way, but they may also have meanings that are unrelated to real-world things like distance or time. Within multiscale models, components can be grounded differently. At one extreme, all components are grounded absolutely. The dominant perspective of such a model may be physical laws, supported by being on the right side of the Figure 1 scales. At the other extreme, all components use relational grounding. The dominant perspective may be observational-level mechanisms and interactions of living components, motivated by being toward the left side of Figure 1 scales. It is noteworthy that within an epithelial cell culture model, groundings between cells, their environment, and each cell's constituents are relational.

In this article, we present and discuss the above issues with a focus on grounding decisions made while carrying out multilevel, multi-attribute, multiscale modeling and simulation (M&S). Grounding issues do not typically pose problems when a model is narrowly focused on a single aspect*^c ^*of a system (e.g., a pharmacokinetic or gene network model). However, when a model aims to describe multiple system aspects (i.e., different phenotypic attributes), including those that cross scale, grounding problems begin to emerge. Below, we argue that a spectrum of multiscale model classes is needed to understand and appreciate groundings and their consequences. Those models that rely exclusively on absolute grounding will occupy one extreme, while those that rely on relational grounding occupy another. With rare exceptions, all current computational biomedical models use absolute grounding. We suggest that developing and making available model classes that use relational grounding is essential for achieving the three goals in the first paragraph.

### Grounding decisions influence model flexibility, adaptability, and thus reusability

Inductive mathematical models are typically grounded to metric spaces and real world units. Such grounding provides simple, interpretive mappings between output, parameter values, and referent*^d ^*data. Absolute grounding*^e ^*creates issues that must be addressed each time one needs to expand the model to include additional phenomena, when combining models to form a larger system, or when model context changes. Adding a term to an equation, for example, requires defining its variables *and premises *to be quantitatively commensurate with everything else in the model. Such expansions can be challenging [1] and even infeasible when knowledge is limited, uncertainty is high, and mechanisms are mostly hypothetical. Such circumstances occur when the characteristics of a problem place it near the center or on the left side of one or more Figure 1 scales. A model composed of components all grounded absolutely or to the same metric spaces--a physiologically-based pharmacokinetic model, for example--has limited reusability when experimental conditions are different or when an assumption made in the original formulation of the model is brought into question. This issue is expanded upon in the context of the first of two main Examples (Example One) presented below. Reusability is hindered in part because a model grounded absolutely conflates two different models (the physiologically-based mechanistic model and the in silico-to-referent mapping model), which have different uses.

By switching to dimensionless, relational grounding (e.g., see [2]), flexibility and reusability are enhanced. With equation-based models, dimensionless grounding is achieved by replacing a dimensioned variable with itself multiplied by a constant having the reciprocal of that dimension. This transformation creates a new variable that is purely relational. It relies on the constant part of a particular organization. However, when dealing with living, changing systems, identifying a constant part with confidence can itself be challenging.

The components and processes in discrete event, object and agent oriented, biomimetic*^f ^*analogues*^g ^*(discussed in detail in [3]), which are created using object-oriented (OO) programming methods, need not have assigned units. See [4-8] for examples in which each constituent and each component is grounded to a proper subset of other modules and components. Cellular automata, agent-based models*^h ^*(ABM), and actor models [9,10] often rely on relational grounding. Relational grounding enables synthesizing flexible, easily adapted, extensible, hierarchical analogues of the systems they mimic.

### Measures, uncertainty, and system information influence grounding choices

The scientific circumstances of any biomedical research problem can be characterized by indicating an approximate location on the three scales in Figure 1. Most engineering (and many molecular and biophysical) problems are characterized by being on the right side of all three scales. From the perspective of cells, tissues, and organisms, a computer chip design problem would be on the far right side of all scales. Most biomedical research problems (i.e., those that deal with systems having living components) would be characterized as being somewhere between the center and the left side of all scales. Being on the right favors reliance on inductive reasoning and developing inductive models that can be precise, accurate, and predictive: the generators of underlying phenomena are well understood, and precise knowledge about mechanisms is available at all levels of granularity. Furthermore, it is straightforward to obtain ample quantitative data against which to validate or falsify the model. As one moves to the left (i.e., with living systems), uncertainty increases. Conceptual mechanisms are less validated (and therefore less trustworthy) and more hypothetical. Reliance on inductive*^i ^*models requires accumulating networked assumptions, some of which may be abiotic. Those assumptions are woven in by reliance on metric and absolute grounding. Difficulties in falsifying mechanistic hypotheses increase dramatically in moving from right to left in part because directly applicable, reliable, quantitative validation (and falsification) data are lacking or scarce. This point is brought into focus in the second example (Example Two), presented below.

Prior to the advent of OO programming, there was no option but to rely on inductive models and metric grounding even though the objects of study were unique and particular. In moving from right to left, one must rely increasingly on abductive*^j ^*reasoning. Consequently, new model classes that support abductive reasoning are needed. The focus should be more on discovering and challenging plausible mechanisms, and less on making precise predictions. Flexible exploration of the space of plausible mechanisms requires models that use relational grounding.

Further discussion will benefit from specific examples. In the next two sections we present and discuss two multiscale modeling examples. We then return to the discussion to address knowledge embodiment; combining models to form larger models; the multi-model nature of models grounded absolutely; multi-paradigm modeling; facilitating translational research; modeling normal-to-disease transition; providing component autonomy; and synthesis of large composite, multi-module, models. The two examples focus on two very different types of multiscale models. Example One illustrates some of the difficulties in reusing and combining absolutely-grounded, physiologically-based, pharmacokinetic (PBPK) models in a cross-domain scenario to make a more biomedically useful, composite, multiscale model. We show how and why integrating four separately developed, absolutely-grounded, models [11-14] is problematic. We argue that, by integrating relational analogues of the four cross-domain models, a mechanistic, PBPK and pharmacodynamic (PD) model can be developed and validated more easily. Example Two focuses on a well-developed hybrid model (ordinary differential equation (ODE) and ABM) of immune cell trafficking behaviors in the context of response to *M. tuberculosis *[15]. The model enables exploring the linkage between grounding decisions, qualities and their relations, and the availability of data against which the model or a component will be validated. Electing absolute grounding presupposes the availability of specific quantitative data, against which to validate, which can be difficult to come by on the left side of Figure 1. Electing relational grounding presupposes the availability of at least qualitative validation data, which is more often available of the left side of Figure 1.

### Example One: Cross-domain integration of absolutely grounded models in quantitative pharmacology

We present an example to illustrate some of the difficulties in reusing models comprised of sets of (differential) equations that are grounded absolutely. We focus on PBPK models in a cross-domain scenario where the models are grounded absolutely. Using relational grounding does not eliminate these difficulties, but it does mitigate them. For the sake of the discussion, suppose one were to develop a detailed mechanistic, PBPK and PD model to predict the disposition and dynamics of a novel, targeted, monoclonal antibody linked to a toxin, for treatment of a localized malignancy. Suppose further that the monoclonal antibody targets surface antigens on developing malignant leucocytes (for example, rituximab), and as such locally concentrates the toxin (e.g., ^131^I), which in turn potentiates its therapeutic effects.

The problem at hand is complex and involves several modeling perspectives: a) pharmacokinetic considerations and disposition of the toxin-antibody complex, which generally follows antibody kinetics, b) pharmacodynamics of the antibody, with or without the toxin, c) pharmacokinetics and disposition of the released toxin, which generally follows simpler compartmental kinetics, and d) pharmacodynamics and toxicodynamics of the toxin. Because certain measurements, for instance antibody tissue distribution data, will be difficult to obtain in human subjects, it is expected that extrapolation or scaling of results from animal studies will be needed.

One option is to develop empiric models to describe the kinetics and dynamics of the novel drug in humans. Another is to develop a multi-level mechanistic model from scratch using data from extensive human studies. A more attractive option is this knowledge-based approach: integrate existing, validated models from the literature to leverage prior efforts and knowledge. A handful of detailed models have been reported that, together, cover each part of the modeling problem. Here are four.

A: Garg and Balthasar [11] present a detailed PBPK model to predict immunoglobulin-gamma (IgG) kinetics in mice in general, where the influence of neonatal Fc-receptor on IgG clearance and disposition is specifically modeled.

B: Merrill et al. [12] present a PBPK Model for radioactive iodide and perchlorate kinetics and perchlorate-induced inhibition of iodide uptake in humans.

C: Scheidhauer et al. [13] present a biodistribution and kinetic model of ^131^I-labelled anti-CD20 monoclonal antibody IDEC-C2B8 (rituximab) using results from a human dosimetric study.

D: Roberson et al. [14] present a pharmacodynamic model of ^131^I- tositumomab radioimmunotherapy in treating refractory non-Hodgkin's lymphoma; the model includes both an antibody antitumor response and a radiation response.

An ideal strategy for achieving the above task is to integrate the four detailed models to form a mechanistic, PBPK and PD model of the novel therapeutic. Here, we discuss some of the barriers, with a focus on consequences of absolute grounding. All four models are grounded absolutely.

#### Unit of measurement in Example One

When there is a lack of direct measurement unit translation between different models, significant barriers arise when one attempts integration. Case in point: models B, C, and D all describe the amount of (radioactive) iodine in a human body. Model B presents iodine dose in milligram and concentration in nanogram/liter; model C presents administered iodine dose in radioactivity units megabecquerel (one million counts per second) and amount of radioactive iodine distributed in regions of interest per injected dose in units of milligray/megabecquerel; model D presents administered dose in microcurie and mean absorbed radiation in gray (or joule per kilogram). Because radioactive iodine is continuously decaying, there is no simple formula to convert mass of iodine at some time (which presents a mixture of ^131^I and non-radioactive iodine) to its respective radioactivity. Also, because the biological effect of radiation dose is measured by radioactivity divided by tissue mass, there is no direct map from tissue levels (mass of drug per tissue volume) to absorbed radiation dose (energy per tissue mass) of the tissue. Hence, the kinetics of iodine from model B cannot directly inform the dynamics as presented in models C and D.

The above problem can be resolved using relational grounding methods with a series of mapping models to translate to physical units. Start by representing matter in fraction of administered mass. In the kinetics context, map to concentration using the tissue volume. In the dynamics context, map to radioactivity using a probabilistic decay model with respect to the decay half-life, which in turn maps to absorbed dose using tissue mass.

#### Adding a compartment in Example One

The goal of developing model A was to predict distribution of a generic monoclonal antibody, and the thyroid gland was not of particular interest, therefore, the thyroid gland was conflated into Other Tissues. Because the primary toxicity of radioactive iodine is destruction of thyroid tissue, the thyroid gland was explicitly represented in model B. On the other hand, model A represented lymph flow, whereas model B did not. Despite the overwhelming similarity of model structures, integrating them is difficult because there is no straightforward way to "insert" a new compartment or flow path. Doing so would require decomposing Other Tissues into, for example, Thyroid and [Other Tissues - Thyroid]. The new components would need to be parameterized and the resulting model would then need to be refitted. Thus, the parameterized PBPK model cannot be reused easily. In models C and D, subjects received either excessive nonradioactive iodine or perchlorate to block thyroid uptake of radioactive iodine. However, the toxicity of ^131^I was not modeled. Linking model B with models C and D would provide toxicokinetic and toxicodynamic insights. However, again, because in models C and D the distribution to the thyroid was not represented, integrating models B, C and D would require extensive model re-engineering and would require extracting additional, enabling measures from the literature.

Had model A combined relational grounding with modular components, it would be easier to add a new component or replace one component with two. There are several options for simulating continuous flow without specifying an absolute volume. For example, drug flow to each compartment can be simulated discretely using probabilistic functions: the drug has certain probability to reach the target compartment each simulation cycle. When needed, one could use a continual stream of discrete amounts. It would then be straightforward to insert an additional tissue component without reengineering the rest of the model, although it would still require reparameterization.

#### From nested, conceptual model to flattened equation model in Example *One*

Figure 1 of [11] provides a way to visualize the entire model in the context of grounding. It is replicated in a slightly different form in Additional file 1, Fig. S1. Visualizing the model as a graph allows us to collapse and expand some sections of the graph, explicitly representing the hierarchical structure in the whole, prosaic, model and the non-hierarchical structure of its mathematical implementation. The tissue nodes in Additional file 1, Fig. S1 expand into sub-models that show the compartments within each tissue, vascular, endosomal, and interstitial, shown in Additional file 2, Fig. S2. Similarly, Additional file 3, Fig. S3 expands the endosomal compartment to reveal the relationship between the antibody and its receptor, the bound fraction derivation in the paper. The series of figures is intended to provide insight into the modeling process, wherein a prosaic model with some hierarchical depth is flattened into an abiotic, system of equations that is grounded absolutely.

#### Adding another antibody (or molecule) to model A of Example One

If disposition of the two molecules is totally independent, then these same equations can be used. Some parameter and variable value estimates can be obtained from literature. Known and unknown parameter and variable values may be different from those of model A's IgG, which means the fitting and prediction (experiment) structure will be different. In general, however, the equations will have the same form and the composite model simply has twice the number of equations, variables, parameters, etc. Such a composite might be largely uninteresting. If, on the other hand, the new molecule is dependent on some components of the IgG model, then integration becomes more difficult if not problematic. For example, perhaps the new molecule also binds to the neonatal Fc-receptor. In that case, adding the new equations should change many of the values of the parameters and variables used in these equations and require either new equations relating the new molecule to IgG or new terms in some of the equations.

#### Adding to model A another receptor that also binds IgG in Example One

New terms will need to be added to the equations governing the tissue components in which cells express the new receptor. Equations governing the tissues components where the new receptor is not expressed will stay the same. New derivations may be necessary for the relational fraction [un]bound. The resulting composite model would then need to be refit to a larger and perhaps more complex data set.

#### Scaling between species in Example One

Consider scaling antibody clearance in mice in model A to enable human prediction. Model A is grounded absolutely on both concentration (mass and volume) and time, and hence, scaling the mice clearance values (in ml/day/kg) to human clearance values would require applying mass, volume, and time scaling factors to all parameters simultaneously, with each of the scaling factors being imprecise and uncertain. When the scaled, parameterized model gives predictions that deviate significantly from observed values, there is no way to ascertain which scaling factor(s) and/or which scaled parameter(s) is problematic.

The relational grounding approach offers a somewhat simplified alternative. The mass parameter scaling and volume parameter scaling can be done separately from the rest of the model and can be validated independently. Time scaling is more complicated but it can be accomplished by finding an appropriate time-scaling factor for all probability parameters. Each of the scaling factors and/or parameters can be adjusted individually to obtain best similarity. In time, automated scaling, which is feasible with models grounded relationally, will expedite the process.

#### Representing uncertainties in Example One

In equation-based models that are grounded absolutely, variables and parameters are often expressed as precise mathematical values, although there are usually significant uncertainties associated with them. Examples of which are the so-called physiological parameters such as (average) blood flow values in a typical PBPK model. Representing uncertainty within a system of differential equations grounded absolutely is mathematically complex (indeed, entire fields of mathematics have been developed to deal with these issues more holistically). Integrating models from different contexts can require adjustment of parameter values, and that in turn requires that the whole model be refitted. In contrast, in a model using relational grounding, probabilistic functions represent inherent uncertainties conveniently. Further, the causes for being unable to adequately match (or later falsify) a relationally grounded model are made more obvious by the explicit inclusion of probabilistic functions.

An important use when developing a detailed, mechanistic, PBPK/PD model is to assist in the design of first-in-human clinical trials of the novel therapeutics. Another is to predict the clinical disposition and response in patients who have not received the therapeutic. In the above examples, two targeted radioimmunotherapies (^131^I tositumomab and ^90^Y ibritumomab tiuxetan) were marketed, and both required individual, empiric dosimetric studies before the therapeutic regimen could be given. It can be argued that, by integrating relational analogues of the four cross-domain models, a mechanistic PBPK/PD model can be developed and validated more easily. The resultant model would likely reduce--possibly eliminate--the need for the dosimetric study.

#### Take-home message from Example One

Analysis of the example models, particularly Garg and Balthasar [11], in the context of model grounding is intended to shed light on the impact grounding decisions have for the resulting model and its uses. It should be clear that ideal use cases for logically deep, relational, models may be quite different from use cases for flattened, absolutely-grounded, models. The cited papers give clear evidence for the use of this example in quantitative prediction and evaluation. The analysis above provides justification for our claim that flattened, absolutely-grounded, models are not ideal for use cases requiring progressive [iterative] evolution and long lifetime models. Specifically, it would be sub-optimal to rely on these models for the exploration of a wide variety of different experimental contexts because it would be difficult to include additional compartments, antibodies, or receptors, to translate to other species, or to represent composite uncertainties.

### Example Two: Immune cell behaviors in the context of response to *M. tuberculosis*

Example Two is the model discussed in Marino et al. [15]. For reader convenience, we cite three closely related papers [16-18] that use essentially the same model.

Example Two was selected because it is a hybrid model. We describe its unique grounding and its impact on the validation of such models. The example serves to illustrate the distinction between absolute, relational, and metric grounding.

Marino et al. [15] examine the roles of immune effector cell activation and migration in the context of response to *M*. tuberculosis. The model attempts to understand the spatiotemporal dynamics of granuloma formulation via linkage, in a hybrid model, combining a complex system of ODEs (grounded absolutely) to explain immune cell dynamics in lymph nodes (LN-ODE), and an agent-based model (ABM) of granuloma formation in pulmonary tissue. The biological process is complex and involves multiple cell types acting in highly variable spatial domains and across many timescales. The composite model is intended to examine the roles of immune effector cell activation and migration in the context of response to *M. tuberculosis*, which usually entails a granulomatous inflammatory response by the immune system. The goal is to understand how the systems influence each other and give rise to their systemic behavior by explicitly modeling the feedback cycle between the lymphoid and pulmonary components.

#### Linking differently grounded sub-models in Example Two

Of critical importance is the notion that to approximate cross-compartmental dynamics (i.e., to account for immune trafficking between the lymph node and the lung), the two components, each grounded differently, must be linked via concrete mappings in order to produce behavior comparable to that from wet-lab models. So doing requires methods for smoothing discrete outputs from the ABM and discretizing the smooth outputs from the ODE. In the [15] hybrid, these mappings involve (in brief)

i) clustering of the APC inputs to LN-ODE into pulses and

ii) discretization of the T-cell fluxes from the LN-ODE.

These intra-component mappings comprise a fundamental part of the grounding of any model.

The output from the ODE model subsequently fed in to the ABM consists of real-valued units, i.e., fluxes, measured in number of effector immune cells arriving to the lung compartment of the model, for each time step of the ODE integrator. These fluxes are computed based on grounding or parameterization comprised of a set of rate constants describing immune dynamics on various scales, ranging from death and proliferation rates to migration rates. The fluxes are derived from continuous equations (ODEs), but because the ABM is grounded to discrete sets, the precise continuous flux output values are mapped into clusters (in this case, T-cell subsets) for input to the ABM. These discrete "bins" or clusters are distinct, if subtle, qualities for the ABM component. The qualities distinguished are integer increments in the counts for each T cell in the queue and its route into the ABM by a chosen vascular source. Viewing the discretization in this way may not, at first, appear to add anything to our understanding of grounding and validation of simulations. However, this perspective illustrates an oft-hidden assumption/process that takes place in all biological M&S research. All continuous T cell output values are qualitatively labeled as being a member of one of the clusters (integers), and these clusters form the basis for all the discrete computation in the ABM. (Note the inverse of such clustering is often called "soft computing," e.g. fuzzy logic, where naturally discrete qualities are coerced into a smooth quantified spectrum.)

A consequence of the preceding situation is that, in effect, the agent-based model depends intricately on the assumptions made in the ODE model and the discretization of its outputs. Conversely, output (in discrete numbers of cells) from the agent-based lung component needs to respect the continuity of the ODEs describing the lymph node component, which constrains solution "spans" of the ODE system to the time step chosen for the agent-based simulation; in essence, the two components of the hybrid model must be linked by a common timescale, the choice of which likely has important implications for the results obtained.

#### Example Two illustrates how groundings influence sensitivity analyses

Given the inherent complexity of the mechanisms being modeled by the ODEs, the above issues present difficulties with model sensitivity analysis. In order to make the ABM a steadily more realistic description of its referent lung tissue, the other components (perhaps just the LN-ODE) must be similar enough to their corresponding referents (e.g. the lymph node) to provide an adequate simulated environment for validating or falsifying the ABM mechanisms. The variation in the behavior of the ODE, via the discretization and smoothing couplings between them, provides context, including bias and constraint, to the ABM mechanisms, which is especially important because the two are linked in an iterative (positive, negative, or stable) feedback cycle. This means that any subsequent changes in the ABM based on the falsification of a mechanism, the incorporation of new hypotheses or domain knowledge, etc. will likely require re-parameterization or reformulation of the ODE, which may undermine the extensive sensitivity analysis already performed.

To overcome these difficulties, an alternative formulation might be to develop relationally-grounded models of both compartments and link them together as a first step, at least until some degree of model validation is achieved. Employing a relational design from the outset facilitates individual component replacement, limiting any one component formulation solely to its coupling with the others. Any component can be replaced at will as long as the minimal requirement of matching I/O with its neighbors is met. This contrasts with an absolutely-grounded model where it is often very difficult to replace only a single component. After a model using relational grounding has undergone degrees of mechanistic validation, some components could then be replaced by absolutely grounded components to see if the mechanistic insight gained from the relational model translates, i.e. those components for which there exists little or no quantitative data against which to validate. In general, starting with a relational model allows us to progressively iterate from qualitative to quantitative validation. With respect to this specific example, replacing the LN-ODE with an articulated, relational model may have the effect of providing an efficient path, through iterative refinement, to quantitative validation of the hybrid model.

#### Qualitative and quantitative validation issues in Example Two

In a wider context, simulation validation is based on its similarity to a referent system. *Similarity *can be defined on a spectrum, ranging from "qualitative" to "quantitative." With qualitative similarity the attributes of a system (simulation or referent) are fundamentally distinct. Objects will either possess some quality, or they will not. Attributes of the simulation and its referent system are considered similar if they have (almost) the same qualities. In [15], clearance, containment, and dissemination of bacterial objects serve as qualitative attributes that can be ascribed to these experimental systems, and by which any two systems can be compared. With "quantitative" similarity, by contrast, attributes are categorically the same but vary by magnitude and can be compared by some ordering relation (e.g. *less than *or *greater than*). In [15], quantitative measures include (among others) cell number; in a given quantitative scenario, a central question concerns the assessment of similarity between the number of cells of a given type produced by a simulation with the number inferred by some experimental assay of the referent system. Despite the spectrum used to define "similarity," it is always the case that a qualitative description is a prerequisite for quantitative descriptions, in the sense that any quantities defined must relate to one or more qualities. In the [15] LN-ODE, the rate bins and types of T cells recruited to the lung component of the model are qualities within which quantities are defined. Hence, all quantitative models are, in some sense, both qualitative and quantitative. In general, quantitative validation will occur only in the context constrained by the available qualities, explaining why qualitative validation must precede quantitative validation. For example, in [15], the LN-ODE is only qualitatively similar to its referent lymph node in the variables the authors chose to model. Obviously, there are many other attributes of a real lymph node that this system of ODEs does not capture and, likewise, there are many attributes (computational, algorithmic) that are superfluous to the biological mechanisms of, e.g., lymph nodes.

As such, the issue of validation (or lack thereof) should act as an important determinant of what type of model (and therefore, what type of grounding) to employ to model the system of interest. Quantitative descriptions should provide quantitative predictions and, hence, allow quantitative falsification. Further, models grounded absolutely make predictions and allow falsification directly in the units to which they are grounded. Importantly, because their (fewer) attributes are more easily quantified, those predictions will be precise and precisely falsifiable if (and only if) there is quantitative data from the referent with which to compare those predictions. In contrast, qualitative descriptions enable merely qualitative prediction and falsification. Relational models, each component defined only in terms of its neighboring components, are more amenable to qualitative descriptions, expressing qualities not necessarily comparable by order or magnitude. Relational models are capable of expressing quantitative predictions and can be quantitatively falsified. However, such quantitative prediction is more complex with relational models because of the logical depth of such models and the high variation expressible within them.

#### Qualitative and quantitative prediction issues in Example Two

Marino et al. [15] comprises a model meant to make qualitative, mechanistic predictions of interest to biologists, but the choice of employing an absolutely-grounded model component suggests that quantitative predictions are expected from their overall model. However, the authors explicitly concede that the LN-ODE model is difficult to quantitatively validate. This situation questions the usefulness of a quantitative model that is amenable to only qualitative validation and makes only qualitative predictions. If only qualitative prediction and validation are possible, is the development of a quantitative component worthwhile? We suggest the answer might be yes, if there is a clear path from the qualitative predictions to quantitative data against which to falsify those predictions. Having quantitative data that agrees with quantitative predictions provides a degree of validation, but no new knowledge is created. Quantitative falsification is more useful because we learn how and where the knowledge instantiated in the model is inaccurate.

#### Guideline and recommendation based on Example Two

As a general guideline, when quantitative validation data are not available, it is most reasonable to first build a qualitative, relational model, and then qualitatively validate and make predictions that will help design experiments on the referent system (i.e., perform wet-lab experiments), the results of which may then be used to refine the qualities and relations in the model. This process should be iterated until quantitative data is available to help falsify the model and make precise predictions. In doing so, we are moving from left to right in Figure 1. Only at that point does it make good sense to develop a quantitative, absolutely-grounded model.

With an eye to further avenues of research, we note that the overarching biological mechanisms explored by this hybrid model (immune cell priming within the lymph node, immune-cell trafficking between the lung and lymph node, and granulomatous containment of bacteria within the lung) are themselves composite processes whose subcomponents (e.g., at the proteomic or genetic level) encode complex, multiscale processes. Any extension of this hybrid model to account for further complexity (i.e., more complete mechanistic insight) will be unguided without quantitative validation for, at least, the LN-ODE component. Similarly, further development of the ABM component would be unguided were there no qualitative validation. However, Marino et al. [15] do specify qualitative validation from the literature and qualities derived from wet-lab experiments. Only after a significant degree of validation, both quantitative and qualitative, is achieved, should further levels of complexity be explored.

### Knowledge embodiment requires models (synthetic*^k ^*analogues) that are relational

Grounding all the elements of a model absolutely, to real, physical units like meters, seconds, and μg/ml is the standard method for hooking the semantics of a computational model to the conceptual models on which biomedical scientists rely. Such grounding is common because it makes the computation purely mechanical, a black box function that "mindlessly" takes input and transforms it into output. Humans interpreting the I/O of the black box do all the semantic grounding for such a model manually, outside the computational framework. Hence, neither knowledge nor semantics, is embedded in the model. Various computational methods have been invented to improve that reality and to allow embedding knowledge in the machine. Expert systems isolate the semantic grounding at the initial conditions and apply a language grammar to mimic logical reasoning: it is the most obvious example of embedding knowledge into a computation. However, relational databases, cellular automata, artificial neural networks, object orientation, agents, actors, etc. do much the same, albeit for different types of knowledge. These methods represent an implicit embedding of knowledge by mapping computational mechanisms to the hypothesized referent mechanisms.

To illustrate, consider a finite volume method simulation of a fluid that consists of discretized blocks and equations that determine the flux for each block. The equations inside the blocks (black boxes) map to the high-level flow dynamics of the referent fluid. There is no mapping from the model to the molecular interactions that compose the higher-level fluid dynamics. The simulation implicitly contains the conservation of mass and the spatial relations that allow it to stand in for the referent and behave similarly, as specified by the PDEs that describe the high level behavior of both systems. Each volume block is grounded to those around it. This example of implicit knowledge is trivial, however, because the I/O for each volume is grounded absolutely to the same units. That absolute grounding makes the relative grounding of a volume with its neighbors invisible to the users. In order to progressively embed more and more knowledge into a computation, such relative grounding must be made explicit.

A much less trivial, implicit knowledge embodiment can be seen in models programmed using OO programming (OOP) languages. Class, instance, property, and method names, along with their respective data types, provide semantic groundings. OOP mechanisms are more obviously grounded relationally because any two interacting objects may only need to understand each other's I/O. Often objects' I/O are buried deep inside the code and the user never sees that I/O. It is this potential for relational grounding that gives OOP its advantage in complex software engineering and in building simulations of complex systems. For knowledge embedded in a biomimetic, computational system to be useful (especially in a social context like shared model usage, validation, and falsification), embedded knowledge must be visible to the user (which is not the case now). Because the focus is science, the user must be aware of the knowledge and must able to discuss it, rely on it, dispute it, and falsify it, all while understanding the domain over which that knowledge, and hence that model, is applicable.

### Relational grounding facilitates referent knowledge embodiment within computational mechanisms

Significant technological progress has been made on methods for embedding knowledge in software, which is required for achieving our stated goals. The dominant method for explicitly embedding knowledge inside a model is with a markup language (XML), a set of core terms and constraints on how they can be used (XML Schema), and a set of related domain specific terms (ontology). They exist because the most common languages used to implement computational mechanisms are universal in what they can express, so called Turing Complete languages. Any practical procedure one can envision is representable in these languages, which makes it difficult to know what a computational mechanism is doing without very close examination. Computational biology markup languages standardize relationships between the terms. Any computation that adheres to the standard can be trusted to, at least, preserve those terminological relationships. This stability facilitates integration, translation, and validation by allowing domain experts (scientists) to examine the mechanisms to a certain extent, one that stops short of forcing them to also be competent computer programmers. Hence, although implicit referent knowledge embodiment within computational mechanisms can, to a limited extent, be accomplished without explicit ontologies, such embodiment will see limited exposure because it prevents specialization into the technical programming versus domain expertise.

The ability for a domain expert to examine a model without needing computational expertise is as critical to the progressive advancement of multiscale simulation in biology as it was for biomedical scientists to design and perform experiments without becoming experts in laboratory equipment design. Progressing from custom-built experimental apparatus (still a common practice fifty years ago) to standardized lab products allowed scientists to compress inherently complicated methods for subsumption by engineering, production, and validation processes. Doing so freed scientists to build their experiments atop complicated lab equipment (e.g., a cell sorter, a confocal microscope, monoclonal antibodies, transfection reagents, automated DNA sequencer) without needing intricate knowledge of the details of equipment processes that produced the final, measured, experiment output. The same progression must occur within computation, and in particular, simulation using modular, multi-attribute, hierarchical, and heterogeneous analogues of biological systems. The subsumption of custom software (the current state of MSM) by engineering production moves knowledge from the mind of the experimenters into the domain of in silico apparatus. Subsequently, scientists will be enabled to design their work around the new technology and its relationship to their system of study.

### Absolute grounding complicates combining models or modules to form larger models unless all are also grounded absolutely

The component and model integration issues identified above balloon into important choices that must be made by the modeler, in the context of the technical and mathematical detail being considered. Bassingthwaighte et al. [19] present and discuss examples of these issues. To illustrate, consider a typical ordinary differential equation (ODE), defined as:

dx/dt = f(x, u, t), x(t_0_) = x_0_; y = g(x, u, t), where,

t is a Real number such that t ≥ t_0_;

x(t) is an n dimensional real tuple representing the state of the system;

u(t) is an m dimensional real tuple representing the system input;

y(t) is an l dimensional real output;

x_0 _is an n dimensional real vector representing the initial condition.

Now consider another ODE that uses a different ordering parameter s, state description p(s), system input v(p), and output q(t):

$$\text{dp/ds}=\text{f}\left(\text{p},\text{ v},\text{ s}\right),\text{ p}\left({\text{s}}_{0}\right);\text{ q }=\text{ g}\left(\text{x},\text{ u},\text{ t}\right).$$

When considering integrating these two systems to form a sibling (lateral, flat, non-hierarchical), the modeler must find mappings t ~ = s, p ~ = x, v ~ = u, and y ~ = q. Almost without exception, this means finding expressions for each element in some real-world units, an absolute ground. More importantly, integrating the two models when the scales are very different, even if the units are the same, presents technical choices the modeler must make. Those choices impact the behavioral solution the scientist sees. See [1] for details and examples. Again, the typical solution for engineered systems is to ground the entire model in the same, low-level units, a "least common denominator" as it were, effectively flattening the model. Note that there are modeling tools like Ptolemy II [20] that help the modeler make these decisions with much reduced effort [21]; but as Bassingthwaighte et al. [19] make clear, the decisions must still be made.

Grounding to hyperspaces increases flexibility and extensibility. A hyperspace*^l ^*is a composite of multiple metric spaces (and possibly non-metric sets). Grounding to a hyperspace provides an intuitive and somewhat simple interpretive map (e.g., see [22]). Relational and hyperspatial grounding*^m ^*is more intuitive and understandable by the biomedical scientist than are absolute groundings. Phenomena and generators are more distinct, because derived measures will often have hyperspace domains and co-domains, making them more complex as interpretive functions. Hyperspaces are often intuitively discrete, so they do not require discretization. They thus handle heterogeneity better than does a model grounded to a metric space. The High Level Architecture (i.e., IEEE standard 1516-2010) and federated systems for distributed computer simulation systems provide for hyperspace grounding. Their focus is to define interfaces (boundary conditions) explicitly so that components adhere to a standard for such interfaces.

Hybrid versions of grounding methods are also possible. Some models can be synthesized by plugging together components consisting of simpler models. For example, in [23], the outputs of metrically-grounded, equation-based models of subcellular molecular and cell cycle details contribute to discrete rules used by cell level agents.*^n ^*Such coupling makes them somewhat relational because not every component must be connected to every other component or grounded to the same data types throughout. However, their synthesis will depend in a fundamental way on their grounding, as in [24] and [25]. Example Two above is a hybrid model. Models adhering to the High Level Architecture and similar standards can be considered hybrids, because their sub-models can integrate in a variety of ways, either relationally or absolutely.

### Absolute grounding can simplify integration by forcing common units and, hence, a common integration target, but context change may require model reengineering

As discussed by Hunt et al. [3], absolute grounding makes the model very fragile to changes in referent context, where context means the particular situation and conditions of the experimental data used to produce the model or in which the model will be used. Inductive, equation-based models are typically grounded to metric spaces because they are induced from particular experiments. They are often fragile to changes in context. This is the case for both Examples One and Two above. The expanding network of assumptions on which the inductive biological model depends makes them increasingly fragile to context change as one moves from right to left in Figure 1. This means they are unlikely to be reused (in fact, the overwhelming majority are not reused). However, an important, scientific motivation for interest in multi-attribute, multiscale, hierarchical, biomimetic models is to explore new uses, such as the consequences of interventions, specifically therapeutic and environmental, or of abnormalities (such as tumorigenesis). For the referents, such interventions often alter context, and these alterations need to be accounted for by the models.

When one changes the environment and experimental conditions of a wet-lab system (primary tissue explants, for example) from *A *to *B*, the living constituents simply adapt. An inductive, absolutely-grounded MSM that is parameterized and has undergone validation against phenomena measured in context *A *may need to undergo considerable reengineering and reparameterization in order to validate against the altered phenomena measured in context *B*. Analogues that use relational grounding can be easily adapted to function in a new context, but the in silico-to-referent mappings will change.

### Models grounded absolutely are implicitly multi-model and making that explicit can be useful

Consider a large, multiscale, ODE model of cancer growth (under specific experimental circumstances) that is grounded absolutely. It is an accretion of several model components, including 1) one or more equations ideally describing a phenomena/mechanism of interest 2) a set of features/aspects hypothetically (conceptually) generated by (usually incomplete) understanding of a specific referent system, and 3) the corresponding measurement units for 2), conceived to provide a precise, quantitative mapping to the referent system. The conceptual models are grounded to the biology via the literature and may contain bias or questionable assumptions from earlier modeling and (likely) experimental efforts. Good science requires that these different models be specifically identified. Such model accretion reduces flexibility and limits reuse. An optional approach to model the same cancer growth (e.g., [26]) would be to first conceptually map the components of specific referent system features with actual, concrete software objects and spaces that have execution protocols. Such a system would be grounded relationally. Actual measures (as opposed to hypothetical assignments) could be taken of phenomena generated during execution. Most of the latter measures would be grounded metrically, but their units would be arbitrary. Finally, quantitative mapping models would be required and used to relate measures of in silico phenomena to real measures of referent phenomena. Such model separation increases flexibility and encourages reuse. Examples are provided by [5-8,17,26-28].

### Multi-paradigm*^o ^*modeling requires both hyperspatial and relational grounding

Even in the simplest case of multi-paradigm modeling, integrating a discrete event sub-model with a discrete time (e.g., ODEs) sub-model, mappings must be made between the components so that the system as a whole behaves appropriately. Example Two above illustrates such mappings. When metric grounding is preferred, the most expedient route is absolute grounding. In that common case, the units of the entire system provide the "least common" grounding so that the events and the I/O of the discrete time components will relate. This effectively flattens hierarchical models and provides a single, ideal, paradigm (that of an approximation to a continuous system) to which all components relate (e.g., Example Two, the hybrid model discussed above). However, MSMs in which each component can consist of internal mechanisms that are unrelated to (can be independent of) the internal mechanisms of other components will require relational grounding. To preserve hierarchy (avoid flattening), hyperspatial grounding will also be required.

### Biomimetic analogues designed to facilitate translational research and development must have long lifecycles

Cell culture models have attributes for which human counterparts are believed to exist. They also have some attributes for which there are no human (or whole organismal) counterparts. This is true of all model-referent relationships. Conceptual mappings from one wet-lab model to another or from a wet-lab system to human referents can be supported by validation evidence, yet they are difficult to falsify: falsification, not validation, leads to new knowledge. One relationally grounded analogue can be morphed into another. Park, et al. [6] provides an example. That morphing stands as a model of the conceptual mapping: it can be directly challenged and falsified (or not). Biomimetic analogues designed to support translational research are expected to evolve, to be iteratively revised following experimental challenge [3,29]. We expect them to be expanded to become more realistic and trustworthy, and thus more scientifically useful. Their limitations will also become better understood, which increases the believability of the model as long as it is used within its limits. At the same time, it points out what conditions are perhaps not being treated properly in the model. Consequently, the models must be adaptable and extensible, and this (we maintain) requires that they employ relational grounding, like their referents. Those that do can possess long lifecycles. They will help us understand what does and does not translate from one wet-lab model to another as well as what can actually transition from bench to bedside. Some will mature to become virtual tissues and organs, to be used as components in virtual patients [3]. We suggest that virtual biology components begin as relational analogues and remain so to the degree feasible, and that separate model-to-referent mapping models be developed in parallel.

### Exploring mechanisms of normal-to-disease transition requires model components that use relational grounding

Multi-attribute, MSMs are expected to help achieve exploitable insight into normal-to-disease transitions and facilitate discovery of new treatment options. Normal-to-disease transitions may involve changes in how components at multiple levels interact. Multilevel structural changes can occur. Those circumstances place us on the left side of Figure 1 scales where inductive models grounded metrically may not be up to the task. On the left side in Figure 1, many alternative mechanistic scenarios need to be explored and challenged. Having components grounded absolutely makes exploration of that changing mechanism space problematic. Reliance on relational grounding simplifies mechanism exploration and makes the process more intuitive.

### Components in composite, multi-attribute, biomimetic modules and models need some autonomy: relational grounding facilitates providing component autonomy

All models have a degree of articulation*^p^*, which is the extent to which the model consists of distinct parts, modules, or components. Bassingthwaighte et al. [19] discuss the distinctions in the context of multiscale, mathematical models that rely on absolute grounding. An ODE model, for example, can be analyzed into parameters, variables, terms, etc. Some distinct components of a cellular automaton model are its transition rules for each cell, and the states held by cells. A model's articulation is the extent to which the components are encapsulated and the internal dynamics of the components are independent of those of the other components. This concept extends beyond the typical OOP encapsulation of state and behavior into activity, and is critical for composite, biomimetic models. When a component does not need any other components to enable initiating and maintaining its own run-time, then that component is autonomous. Mammalian cells can be autonomous in vitro. Scientifically useful in silico models, of these referent systems will likewise need to possess autonomy.

Tissues and organs are highly articulated systems. The components in a highly articulated model of one of those systems will need to be quasi-autonomous. That means that they can be effectively replaced by other components. Such a situation is achieved by specifying the I/O requirements of the components in ways consistent with biology. If module I/Os are specialized and tightly coupled to the other components and modules in particular and unique ways, then the model can be called a "composite" or "articulated" model from an engineering standpoint. However, from a practical perspective, it remains monolithic. One cannot easily remove (unplug) a component and use it elsewhere. It is straightforward to isolate primary epithelial cells, separate them, and study them as tissue culture models. M&S requires computational analogues possessing similar capabilities, since, taking a long-term, view, we want our analogs to be "alive," to whatever extent is possible using computation, so that they are as similar as possible to their referent systems. After all, biology is the study of life, and so biological models must mimic attributes of life; indeed, "biomimetic" is not a catachresis.

The spectrum of model articulation issues is orthogonal to those of absolute vs. relational, and metric vs. hyperspatial grounding. The extent to which a component is autonomous is handled by the clear specification and maintenance of component use cases*^q ^*(aspects; phenotypic attributes) or, collectively, the component's phenotype. Autonomy can be established regardless of how the model is grounded, but only when targeted phenotypic attributes are clearly defined. For example, one might argue that a purely relational model, where every component's I/O is meaningful only in the context of the other components with which it communicates, lacks any autonomy. That will be the case as long as there is only one use case for that component and a single use case for all connected components, or as long as that particular, specific organization of components is unique and no other arrangement makes sense/is needed. However, if even one of the components has multiple use cases (multiple plausible configurations; may exhibit different attributes, e.g., when stressed in different ways), then the degree of autonomy of that component (and all those connected to it) increases. Complete autonomy for a component is achieved in the limit as phenotype expands, regardless of how the component is grounded. A Madin Darby canine kidney (MDCK) cell culture can have a huge number of phenotypic attributes (use cases), as can each cell within. Hence, a scientifically useful, in silico analogue of MDCK cell cultures must have a large number of specified use cases. To enable that, the composite cells in the analogue (and components and modules therein) must possess a high degree of autonomy to enable a myriad of specified use cases and biomimetic attributes.

### A composite model (aka, MSM) is a graph of components integrated by I/O edges; relational grounding enables synthesis of large composite (multi-module) models

Composite models can be understood as graphs of black (or gray or transparent) box vertices integrated by I/O edges. The desired or targeted biomimetic attributes for each component dictates I/O types. If two components are grounded differently, there must be integration logic to map the I/Os of the two components. Even in the case where the two components are grounded absolutely and metrically, there must be component intermediaries (e.g., the "scaling translators" in [30]) that map one component's output to another's input. Hence, explicitly handling the edges between the black boxes, as methods for mapping I/O, is a best practice no matter what type of model one constructs.

An additional issue to consider is the extent to which (seemingly) purely technical components are exposed to (integrated with) the modeling layer. There are many models where various technical components must be chosen depending on the details of the model. For example, when a system of ODEs becomes "stiff", i.e., solver instability occurs, even with simple but pathological equations like y = (x^2 ^- 3x)/(x - 3.001), which is erroneously solved by Mathematica. Such examples provide further evidence that the explicit design of inter-component mappings is a best practice when developing composite, biomimetic models.

## Conclusions

In this article, we have reviewed and defined model grounding concepts, highlighted their spectrum, and explained when and how modelers should incorporate different types of grounding (or combinations thereof). We have discussed two literature examples of complex models from somewhat different domains but share a common thread in that each could benefit from detailed grounding considerations. We are led to the belief that if we accept that explicit I/O mapping is a best practice when developing composite biomimetic models, and that relational grounding of composite models forces development of such methods, it becomes clear that relational grounding is a robust and preferred modeling method. However, relational grounding is inappropriate when the model being considered is, naturally, monolithic or non-articulated, i.e., when all the components are fundamentally dependent on the structure of the I/O of other components. The latter is often the case when dealing with purely mathematical models. We maintain that grounding issues should be addressed at the start of any MSM project and should be reevaluated throughout the model development and refinement processes. We offer a general guideline. When validation data are not available, it is most reasonable to first build a qualitative, relational model, and then qualitatively validate and make predictions that will help design wet-lab experiments on the referent system, results of which may then be used to refine the qualities and relations in the model. This process should be iterated until quantitative data are available to help falsify the model and make precise predictions. As we do so, we move from left to right in Figure 1, and approach models that more closely represent biological processes.

## List of abbreviations used

AB: agent-based; ABM: agent-based model; I/O: input-output; IgG: immunoglobulin-gamma; LN-ODE: lymph nodes ordinary differential equation; MDCK: Madin Darby canine kidney; M&S: modeling and simulation; MSM: multiscale model (MSM); OO: object-oriented; OOP: OO programming; ODE: ordinary differential equation; PBPK: physiologically based pharmacokinetic; PD: pharmacodynamic; XML: markup language (XML)

## Competing interests

The authors declare that they have no competing interests.

## Authors' contributions

With input from coauthors, TNL developed and wrote Example 1 and ADG developed and wrote Example 2. GEPR contributed original ideas and review insights. CAH and GEPR provided content for early Review drafts. CAH managed iterative refinement of the Review from input provided by coauthors; he synthesized the final version from input provided from coauthors. All authors read and approved the final manuscript.

## Endnotes

### Glossary of key technical terms

*^a ^*absolute grounding: variables, parameters, and I/O are in real-world units like seconds and meters

*^b ^*relational grounding: variables, parameters, and I/O are in units defined by other components of the model. For example, if one component's output is in the set {form_lumen, elongate, bifurcate, branch, form_cleft} and a receiving component accepts elements in that set as its input

*^c ^*aspect: the perspective taken when an analogue is observed; one of many functional effects that result and can be observed when an analogue executes

*^d ^*referent: the system, material, or process represented by a model or model component. It is the real system (subsystem) to which a model (or component) refers. Example: consider the ODE term CELung for concentration of antibody in the endosomal compartment of lung tissue in Example One. The term is the model component and its referent is that concentration in the real tissue.

*^e ^*metric grounding: variables, parameters, and I/O are in subsets of metric spaces. For example, when a parameter takes real number values in the range [-1.0, 1.0]

*^f ^*analogue: anything that is analogous or similar to something else, and that exists and operates in isolation even in the absence of a referent; a system that has aspects and attributes that are similar to those of a referent system; a biomimetic model implemented in software that, when executed, produces phenomena that mimic those of the model's referent

*^g ^*agent-based: something formulated with or built up from agents; [in agent-based modeling] a model designed for simulation in which quasi-autonomous agents are key components

*^h ^*biomimetics is the study of the structure and function of biological systems as models for the design and engineering of materials and machines, in this case computational models. It is often regarded as being synonymous with biomimicry, biomimesis, biognosis and biologically inspired design.

*^i ^*induction: arrival at a conjecture (universal conclusion) based on a pattern observed in many particular cases; generalization: reasoning from detailed facts to general principles; generalization drawn from patterns in observed data

*^j ^*abduction: arrival at conjectures based on a pattern observed in one or a few particular cases; construction of hypothetical speculations (consistent with current knowledge) about the process by which an outcome (phenomenon) came to be, where the hypotheses are all equally reasonable as long as they lead to the outcome; arrival at a conjecture (hypothesis) that would, if true, explain the relevant evidence

*^k ^*synthetic analogue: an analogue system constructed from extant, autonomous components whose existence and purpose are independent of the model they comprise; one formed specifically by combining elements, often varied and diverse, so as to form a coherent whole

*^l ^*hyperspace: a set, X, of sets, x_i_, where each constituent set x_i _may or may not be a subspace of some metric space. For example, the three element set {x_0_, x_1_, x_2_} where x_0 _= {A, B, C}, x_1 _= [-1.0, 1.0], x_2 _= {red, blue, green} is a hyperspace

*^m ^*hyperspatial grounding: grounding to a hyperspace

*^n ^*agent: [technical] an object within an OO program that can schedule its own events [within an analogue: it is quasi-autonomous; it senses and is part of its environment; it pursues and can revise an agenda within a larger script; it is identifiable by an observer as a cause of an effect; its attributes and actions may be designed to represent biological counterparts, whereas others will deal with issues of software execution]

*^o ^*multi-paradigm model: A model that integrates more than one type of computational framework. (cf. http://en.wikipedia.org/wiki/Multiparadigm_programming_language) For example, when a model combines an expert system with several fluid dynamics models

*^p ^*articulation: the extent to which the model consists of distinct, interconnected parts or components; the extent to which components are encapsulated and their internal dynamics are independent of those of the other components

*^q ^*use cases: the aspects of the referent that the model intends to mimic or represent; how and for what purposes the model will be used (simulation scenarios). A component's or model's phenotype: the set of all targeted attributes.

## Supplementary Material

Additional file 1 **Figure S1, referred to in the text**. Click here for file

Additional file 2 **Figure S2, referred to in the text**. Click here for file

Additional file 3 **Figure S3, referred to in the text**. Click here for file
