# Supplementary material for: Relational grounding facilitates development of scientifically useful multiscale models
Source: Theor Biol Med Model. 2011 Sep 27;8:35. doi: 10.1186/1742-4682-8-35 (PMC3200146; doi:10.1186/1742-4682-8-35)
Supplement: Additional file 1 — Figure S1, referred to in the text. [file 1742-4682-8-35-S1.PDF]

Supplemental Material To:

Relational Grounding Enables Scientifically Useful Multiscale Models

C. Anthony Hunt, Glen E.P. Ropella, Tai ning Lam, and Andrew D. Gewitz

Referred to under Example One

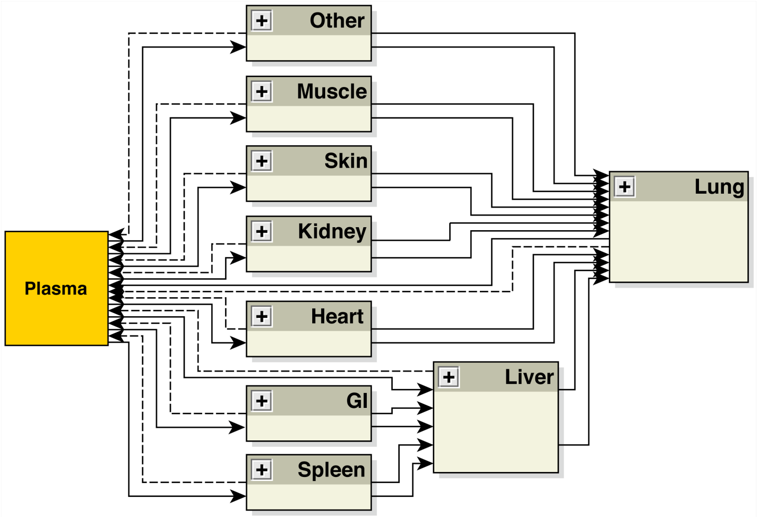

Figure S1. Redrawn Figure 1 from [Garg and Balthasar] showing the central roles of the Plasma and Lung components and emphasizing the separable flow paths for each component. Dashed arrows are Lymph Flow. Solid arrows are Plasma Flow.
