# Supplementary material for: Relational grounding facilitates development of scientifically useful multiscale models
Source: Theor Biol Med Model. 2011 Sep 27;8:35. doi: 10.1186/1742-4682-8-35 (PMC3200146; doi:10.1186/1742-4682-8-35)
Supplement: Additional file 2 — Figure S2, referred to in the text. [file 1742-4682-8-35-S2.PDF]

Supplemental Material To:

Relational Grounding Enables Scientifically Useful Multiscale Models

C. Anthony Hunt, Glen E.P. Ropella, Tai ning Lam, and Andrew D. Gewitz

Referred to under Example One

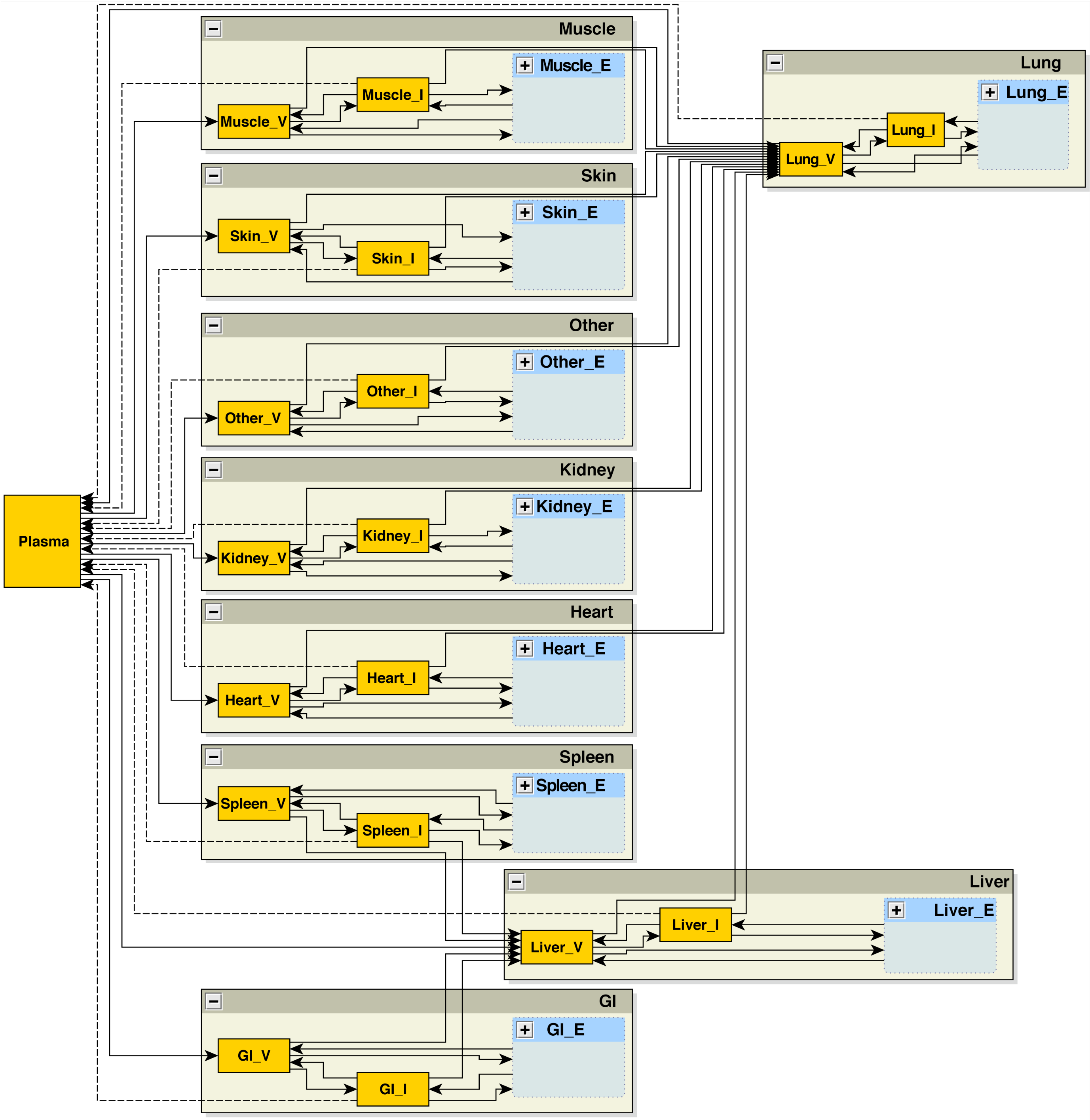

Figure S2. Graph representing the model equations (2)-(11) in the context of the integrated model including Figures 1 and 2 of [Garg and Balthasar]. Figure S3 shows the fully expanded graph of the model. Although the mathematical model and programmed implementation is fully flattened (non-hierarchical), the conceptual model as laid out in the paper and diagrams indicates the hierarchical structure in this graph. Each tissue breaks out into four distinct sub-models: Plasma, Lung, Liver, and Other. The Other of equations (9)-(11) indicates that all the other tissues are modeled the same: Muscle, Skin, Heart, GI, Spleen, and the "Other" in the diagram. The influence of each intra-tissue compartment on the terms in equations (2)-(11) is represented in this graph.
