# Supplementary material for: Relational grounding facilitates development of scientifically useful multiscale models
Source: Theor Biol Med Model. 2011 Sep 27;8:35. doi: 10.1186/1742-4682-8-35 (PMC3200146; doi:10.1186/1742-4682-8-35)
Supplement: Additional file 3 — Figure S3, referred to in the text. [file 1742-4682-8-35-S3.PDF]

Supplemental Material To:

Relational Grounding Enables Scientifically Useful Multiscale Models

Referred to under Example One

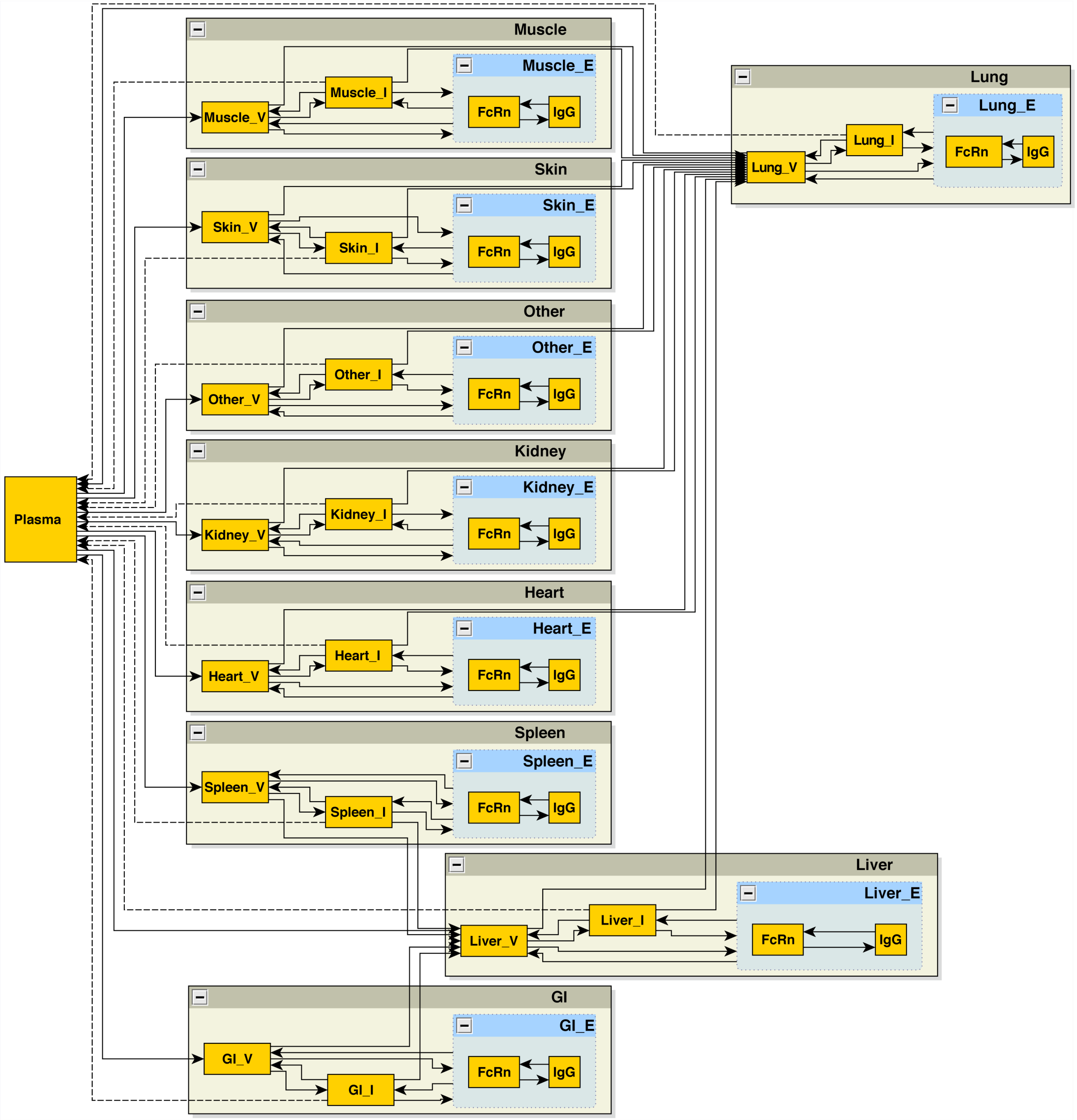

Figure S3. Graph representing the whole, flattened, model including equations (12)-(14), along with the derivation of bound and unbound fraction of antibody. The tissue nodes in Figure S1 expand into sub-models that show the compartments within each tissue: vascular (_V), endosomal (_E), and interstitial (_I), shown in Figure S2. This is the fully flattened graph, reflecting the RcFn receptor interactions with the IgG antibody in the context of the intra-tissue compartments, in turn, in the context of the whole model tissue graph.
